# Effects of primary care clinician beliefs and perceived organizational facilitators on the delivery of preventive care to individuals with mental illnesses

**DOI:** 10.1186/s12875-017-0693-2

**Published:** 2018-01-12

**Authors:** Bobbi Jo H. Yarborough, Scott P. Stumbo, Nancy A. Perrin, Ginger C. Hanson, John Muench, Carla A. Green

**Affiliations:** 1 Kaiser Permanente Northwest Center for Health Research, 3800 N. Interstate Avenue, Portland, OR 97227 USA; 2 Johns Hopkins School of Nursing, 525 N. Wolfe Street, Baltimore, MD 21205 USA; 3 Oregon Health & Science University, 3181 S.W. Sam Jackson Park Road, Portland, OR 97239 USA

**Keywords:** Primary care, Prevention, Mental illness, Schizophrenia spectrum disorder, Bipolar disorder, Preventive services, Clinicians

## Abstract

**Background:**

Although many studies have documented patient-, clinician-, and organizational barriers/facilitators of primary care among people with mental illnesses, few have examined whether these factors predict actual rates of preventive service use. We assessed whether clinician behaviors, beliefs, characteristics, and clinician-reported organizational characteristics, predicted delivery of preventive services in this population.

**Methods:**

Primary care clinicians (*n* = 247) at Kaiser Permanente Northwest (KPNW) or community health centers and safety-net clinics (CHCs), in six states, completed clinician surveys in 2014. Using electronic health record data, we calculated preventive care-gap rates for patients with mental illnesses empaneled to survey respondents (*n* = 37,251). Using separate multi-level regression models for each setting, we tested whether survey responses predicted preventive service care-gap rates.

**Results:**

After controlling for patient-level characteristics, patients of clinicians who reported a greater likelihood of providing preventive care to psychiatrically asymptomatic patients experienced lower care-gap rates (KPNW γ= − .05, *p* = .041; CHCs γ= − .05, *p* = .033). In KPNW, patients of female clinicians had fewer care gaps than patients of male clinicians (γ= − .07, *p* = .011). In CHCs, patients of clinicians who had practiced longer had fewer care gaps (γ= − .004, *p* = .010), as did patients whose clinicians believed that organizational quality goals facilitate preventive service provision (γ= − .06, *p* = .006). Case manager availability in CHCs was associated with higher care-gap rates (γ=.06, *p* = .028).

**Conclusions:**

Clinicians who report they are likely to address preventive concerns when their mentally ill patients present without apparent psychiatric symptoms had patients with fewer care gaps. In CHCs, care quality goals may facilitate preventive care whereas case managers may not.

**Electronic supplementary material:**

The online version of this article (doi: 10.1186/s12875-017-0693-2) contains supplementary material, which is available to authorized users.

## Background

People with mental disorders are more likely than the general population to die prematurely [1]. Disparity in life expectancy, especially among people with serious mental illnesses like schizophrenia and bipolar disorder, is largely attributable to preventable chronic conditions [2], sometimes caused or exacerbated by higher rates of behavioral risk factors [3–5] or by the metabolic side effects of antipsychotic medications sometimes used to treat these illnesses [6–8]. Though individuals with mental illness diagnoses appear to receive preventive services at rates similar to persons without these diagnoses [9], screening for certain conditions, including colorectal cancer or osteoporosis, and receipt of certain services like influenza vaccinations remain low, particularly among individuals receiving care in federally qualified health centers [9]. Rates of annual screening of cardiometabolic risk factors among people taking antipsychotics [10] are also suboptimal, even in integrated care settings, despite clinical screening guidelines that have existed for more than a decade [11], and evidence that people with serious mental illnesses are at significantly increased risk for metabolic syndrome [12], and cardiovascular disease and related mortality [13].

Past research has used qualitative and survey methods to identify patient and clinician perspectives on barriers to primary care among those with serious mental illnesses. Patient-level barriers identified include psychiatric illness severity [14]; cognitive and communication limitations [15]; fears of having a serious illness or dying [16]; belief that illnesses will resolve without medical care, lack of knowledge of where to seek treatment, doubt that treatment will be effective, and shame [17]; lack of resources [18, 19]; and logistical issues (e.g., time and transportation). Considerably less work has identified clinician- or organizational-level barriers. Perceived barriers include negative biases [15], difficulty coordinating care [14] across separated primary and mental health care systems [15, 19], and lack of basic primary care training among mental health providers who may be de facto primary care providers [19]. Even fewer studies have directly tested the relationship between clinician and organizational factors and observed rates of preventive services. One group found that low rates of metabolic testing were more strongly associated with patient characteristics and frequency of visits than with clinician and practice characteristics (e.g., care setting location, type, size; colocation of medical and mental health services) [20]. Very little is known about the role that other organizational characteristics, including team-based care [21, 22], outreach related to preventive care use [23, 24], and organizational cultures conducive to quality care [25] may play in preventive service completion among this population.

As part of a larger project designed to assess and compare overall preventive service use patterns among individuals with and without mental illness diagnoses, the current study assessed whether specific clinician characteristics, behaviors, and beliefs, and clinician-reported organizational characteristics, predicted delivery of preventive screening and care to individuals with mental illnesses. The project included two large samples of clinicians representing a diverse set of health care settings. As in the parent study [9], we used patient preventive care gaps (needed preventive services which were not completed; see Yarborough et al. [9] for additional detail) derived from electronic health records (EHR), to assess 12 recommended preventive activities [26]. To our knowledge, this is the first study to link clinician and organizational predictors of preventive service delivery to a broad array of EMR-derived preventive service outcomes among individuals with mental illnesses.

## Methods

### Settings

Settings were Kaiser Permanente Northwest (KPNW) and federally qualified health centers and safety-net community health clinics (CHC) affiliated with the OCHIN health information technology network. KPNW is a not-for-profit, group-model, integrated health system that provided care to approximately 500,000 members in Oregon and Washington during the study period (2012–2013). KPNW members are representative of the service area in terms of age, sex, and race/ethnicity [27]. OCHIN provides a common EHR platform for its member health care systems enabling EHR-based research across these settings. Thirty-four OCHIN-affiliated health systems met eligibility criteria for recruitment to complete the primary care clinician survey (i.e., served adult patients, had an established EHR system by May 1st, 2012). Of those, 27 organizations representing 107 CHCs in California, North Carolina, Ohio, Oregon and Wisconsin agreed to participate in the clinician survey.

### Clinician sample

Primary care clinicians from participating organizations were invited to complete a survey if they had been employed ≥ 2 years with their organization, had ≥100 empaneled patients, and ≥10 patients with qualifying mental illness diagnoses (schizophrenia spectrum, bipolar, anxiety, and major depressive disorders). Qualifying clinicians (*n* = 489) were mailed a study invitation letter and a chocolate bar. A week later an email followed, which contained an embedded link to the survey with a unique identifier for each clinician. Non-respondents were telephoned 1–2 weeks later. Two hundred forty-nine surveys were completed, a 51% response rate overall (61% of KPNW clinicians and 45% of CHC clinicians sent a survey link).

### Patient sample

Patients empaneled to clinician survey respondents with ≥ two recorded instances of a qualifying mental health diagnosis (schizophrenia spectrum, bipolar, anxiety, and major depressive disorders) were included if they were ≥19 years old and had ≥ one health care visit in 2012 or 2013. Transgender and unknown gender patients were excluded because preventive service recommendations are gender-specific. Also excluded were patients with ≥ two instances of a diagnosis of a serious cognitive or developmental disability and those with a documented preference to be excluded from research. The Kaiser Permanente Northwest Institutional Review Board approved and monitored the study; a waiver of informed consent was granted for analysis of patient-level EHR data, clinicians consented to study participation by returning a survey.

### Primary care clinician survey

A brief, online clinician survey was developed to identify barriers or facilitators of preventive care delivery (see Additional file 1). To inform survey development, formative, semi-structured interviews with clinicians in KPNW (*n* = 15) and CHCs (n = 15) were conducted. Interviews addressed clinicians’ experiences providing primary care to people with serious mental illnesses and how those experiences differed from experiences with other patients, assessments of the relative importance of preventive medicine topics, approaches to and comfort with conversations about prevention and risk factors with this population, and perceived effectiveness of those conversations. Themes from the interviews were used to construct the clinician survey.

### Clinician and organizational variables from the survey

#### Clinician behaviors, beliefs, characteristics

The survey asked clinicians about their experience, knowledge, and comfort interacting with patients experiencing serious mental health symptoms. We measured clinicians’ beliefs about how health-related recommendations could affect patients’ mental health, the degree to which stability of mental health affected clinicians’ willingness to make recommendations about preventive services, and the amount of additional support patients with serious mental illnesses required to be successful with preventive care. We also measured clinicians’ beliefs about whether or not their patients with serious mental illnesses prioritized preventive care, their confidence that these patients would follow their recommendations, and how that level of confidence compared to their confidence in other patients’ ability to follow through.

We asked clinicians to report how often they consulted with mental health providers about their patients’ physical health, and about their likelihood of making eight specific recommendations: smoking cessation, improving diet/nutrition, increasing exercise, reducing alcohol/drug use, getting a mammogram, getting laboratory work, getting a flu shot, and completing colon cancer screening. Specifically, we asked how likely clinicians were to make these recommendations when they encountered patients who were not experiencing significant mental illness symptoms (i.e., were psychiatrically asymptomatic), and how much less likely they might be to make the same recommendations when patients are experiencing acute symptoms. Finally, we asked clinicians to describe their gender, race, ethnicity, age, and years practicing medicine. Most survey responses were rated on 5-point Likert-type scales. Gender and race (white/non-white [includes Hispanic]) were dichotomized; years practicing medicine (post-training) was a continuous measure.

#### Clinician-reported organizational measures

Clinicians reported on a number of characteristics of the organizations in which they practiced, including: availability of behavioral health specialists, case managers, care navigators, health coaches, and peer-provided services to assist patients; use of a team-based care model; co-location of medical and mental health services; community outreach services; or Assertive Community Treatment programs.

We assessed the degree to which several potential care facilitators (e.g., use of mobile prevention services, co-location of laboratory services, EHR best practice alerts) and barriers (e.g., lack of time, insufficient reimbursement, lack of patient interest, acuity of patient concerns in the visit) affected delivery of health promotion and disease prevention. We also assessed the degree to which the clinician’s practice setting emphasized staying on time with appointments, patient satisfaction, a patient-centered approach to care, cost-effectiveness, and care quality. Finally, the survey included a measure designed to elicit the degree to which respondents believed that clinician screening goals imposed by the organization (i.e., targets associated with process of care performance measures), affected the likelihood of providing preventive care to individuals with serious mental illnesses. The clinician survey is available upon request.

### Electronic health record-derived variables

#### Main outcome: preventive care-gap rate

EHR data was used to examine needed preventive care for 12 preventive services: obesity measured by calculated body mass index (BMI), hypertension, dyslipidemia (lower-density lipoprotein), diabetes (Hemoglobin A1c or fasting plasma glucose), tobacco use status, evidence of annual influenza vaccine, pneumococcal vaccine (aged 65+), colorectal cancer screenings (aged 50–75), and, for women only, screening for osteoporosis (aged 65+ who have ever received a bone density test), chlamydia (aged 16–24), breast cancer (aged 50–74, mammography), and cervical cancer (aged 24–64 with at least one Papanicolao (pap) test in prior 3 years). Both KPNW and the CHCs specifically used a panel support tool that took into account patient characteristics (e.g., age, gender, past medical history) to determine needed preventive services, and which flagged patients with incomplete services (“care gaps”). Using the care gaps, each of the 12 preventive services were dichotomized as 0 (up to date/completed) or 1 (out of date/not completed). The proportion of needed preventive care (care-gap rate) was computed for enrolled patients by dividing the number of out-of-date services for each patient by the number of eligible preventive services, and multiplying by 100.

### Statistical analyses

The main analyses were conducted using multi-level regression in HLM 7 [28] to account for the nesting of patients within clinicians. A Poisson linking function accommodated the skewed distribution of care gaps. Samples from KPNW and the CHCs were analyzed separately because the settings used slightly different approaches for identifying patients with overdue preventive services. Responses from clinicians at KPNW and CHCs were compared using chi-square and t-tests to assess differences in clinician and organizational characteristics on survey responses of interest.

We hypothesized that, after controlling for patient-level characteristics, clinician characteristics (female gender [29], years practicing medicine [30]) and certain behaviors (e.g., consulting with mental health clinicians, providing care to psychiatrically asymptomatic patients) as well as organizational facilitators (e.g., access to case managers or navigators, colocation of services, emphasis on quality) would be associated with better care (i.e. lower care-gap rates), whereas endorsing the belief that patients with serious mental illnesses lack interest in prevention would be associated with higher care-gap rates.

We tested each independent variable of interest separately in a model containing all covariates before finalizing each model. The following patient-level covariates were included: diagnosis, age, gender, race, ethnicity, Medicaid status, Medicare status, physical health comorbidity [31], number of primary care visits, and number of non-primary care visits. All variables not significantly related to care gaps in these individual predictor models were excluded from further analyses; dropped variables included: clinician belief that patients lacked interest in prevention, practice emphasis on care quality, team-based care, amount of consultation between medical and mental health clinicians and co-location of medical and mental health or laboratory services. None of these clinician or organizational characteristics showed associations with care-gap rates.

Clinician and organizational variables that were significantly related to the care-gap rate in either the KPNW or CHC sample were included in the final overall model for both samples. These included: clinician gender, years of practice, likelihood of providing care when patients are asymptomatic, availability of case managers, availability of care navigators, and belief that clinician performance measures increase likelihood of providing services.

## Results

Patient panel data was unavailable for two clinicians who returned surveys; the remaining 247 clinicians had an average of 6.8 (SD = 6.8) patients with schizophrenia spectrum disorders on their panel, 17.7 (SD = 11.0) patients with bipolar disorder, 36.3 (SD = 22.0) patients with anxiety disorders, and 90.0 (SD = 43.9) patients with unipolar depressive disorders. See Table 1 for additional results.

**Table 1:** Primary care clinician survey respondent characteristics by site, frequencies and means for model variables^a^ (*N* = 249)

	KPNW *n* = 113 (45.4%)		CHCs *n* = 136 (54.6%)		*p*
	Total N	No. (%)	Total N	No. (%)	
Provider characteristics					
Gender (female)	107	44.9	132	65.9	.001^b^
Race/ethnicity (non-White)	107	24.3	128	15.6	.095
		M(SD)		M(SD)	
Age	104	47.83(8.62)	130	45.44(10.39)	.061
Years in practice	107	15.71(9.11)	132	12.76(10.10)	.202
Provider beliefs/behaviors					
Consult with mental health provider^c^	109	2.22(.79)	132	2.46(.88)	.026^d^
Lack of patient interest affects preventive care delivery^e^	108	1.98(.74)	131	1.85(.73)	.160
Likely to make preventive recommendations when patients not experiencing symptoms^f^					
--Smoking	111	3.59(.64)	136	3.32(.78)	.005^g^
--Diet/nutrition	110	3.30(.71)	136	3.16(.76)	.122
--Exercise	111	3.44(.66)	136	3.18(.80)	.005^g^
--Alcohol/drug use	111	3.44(.75)	135	3.48(.70)	.665
--Getting a mammogram	111	3.62(.52)	135	3.33(.72)	.001^b^
--Getting laboratory work	110	3.62(.54)	136	3.54(.62)	.325
--Getting a flu shot	111	3.62(.57)	136	3.63(.58)	.964
--Completing colon cancer screening	111	3.62(.56)	135	3.19(.81)	<.001^b^
Likelihood of providing care absent symptoms (composite)^f^	111	3.53(.49)	136	3.35(.57)	.010^g^
Organizational characteristics		No. (%)		No. (%)	
Availability of co-located of medical and mental health services	113	41 (36.3)	136	106 (77.9)	<.001^b^
Team-based care approach	113	59 (52.2)	136	116 (85.3)	<.001^b^
Availability of a case manager	113	72 (63.7)	136	86 (63.2)	.937
Availability of a care navigator	113	9 (8.0)	136	60 (44.1)	<.001^b^
		M(SD)		M(SD)	
Co-located laboratory services facilitates care^h^	107	2.23(.95)	131	2.47 (.83)	.046^d^
Emphasis on quality of care^i^	107	1.84(.37)	131	1.54(.50)	<.001^b^
HEDIS screening increases likelihood of delivering care^j^	107	2.54(.57)	132	1.95(.70)	<.001^b^
Provider panel characteristics	Total N	M(SD)	Total N	M(SD)	*P*
Number of patients					
Schizophrenia spectrum disorder	105	3.78(2.08)	131	9.75(7.93)	<.001^b^
Bipolar disorder	113	16.48(6.91)	134	18.75(13.47)	.090
Major depressive disorder	113	121.05(32.59)	134	63.81(34.06)	<.001^b^
Anxiety disorder	113	50.65(19.48)	134	24.25(16.06)	<.001^b^
Total number of patients represented	19,782		14,551		
Mean patient care gap score^k^					
Schizophrenia spectrum disorder	105	16.12(10.65)	131	25.24(9.49)	-
Bipolar disorder	113	16.15(4.34)	134	28.05(7.08)	-
Major depressive disorder	113	14.84(3.02)	134	29.25(5.98)	-
Anxiety disorder	113	17.95(3.04)	134	29.65(6.47)	-

^a^For a description of patient characteristics, please see Yarborough et. al. [9]

^b^p < .001

^c^Coded1 = not at all, 2 = a little, 3 = a moderate amount; 4 = a great deal

^d^ p < .05

^e^Coded1 = not at all/a little, 2 = a moderate amount; 3 = a great deal

^f^Coded 1 = not at all likely, 2 = somewhat likely, 3 = very likely, 4 = extremely likely

^g^p < .01

^h^Coded 0 = not available, 1 = not at all/a little, 2 = a moderate amount; 3 = a great deal

^i^Coded1 = not at all/a little/a moderate amount; 2 = a great deal

^j^Coded 1 = greatly decreases likelihood, 2 = decreases likelihood, 3 = increase likelihood, 4 = greatly increases likelihood

^k^Gap scores were computed differently in each organization therefore statistical comparisons are inappropriate

### Differences between clinician populations

Table 1 presents frequencies and means from the clinician survey for the KPNW and CHC samples. While the survey response rate was higher among KPNW clinicians than CHC clinicians, the CHC sample size was larger such that survey respondents were more likely to practice in CHCs (55% of sample) than KPNW. Respondents were predominantly white (75.9%), had a mean age of 46.5 years (SD = 9.7), and had been practicing medicine post-training for an average of 14.1 years (SD = 9.8). The CHC sample had more female clinicians represented than KPNW (65.9% vs. 44.9%, χ^2^ = 10.65, *p* = .001). The two groups also differed in how likely they reported they were to make preventive care recommendations when their patients with serious mental illnesses were not experiencing significant mental health symptoms. Specifically, KPNW clinicians were more likely than their CHC counterparts to make recommendations about smoking cessation, exercise, mammogram, or colorectal cancer screening (KPNW mean = 3.53, SD = .49; CHC mean = 3.35, SD = .57; F(1245) = 6.75; *p* = .010). KPNW clinicians were also more likely to report that an emphasis on care quality facilitated preventive service provision (mean = 1.84, SD = .37 compared to mean = 1.54, SD = .50 at CHCs; F(1236) = 26.57, *p* < .001), and that quality-related performance goals increased their likelihood of providing preventive care (mean = 2.54, SD = .57 compared to mean = 1.95, SD = .70 at CHCs; F(1237) = 50.0, *p* < .001). CHC clinicians, however, were more likely than KPNW clinicians to report consulting with mental health providers about their patients’ physical health (mean = 2.46, SD = .88; KPNW mean = 2.22, SD = .79, F(1239) = 4.99, *p* = .026); practicing within a team-based care model (85.3% compared to 52.2% at KPNW, χ^2^ = 32.34, p < .001); and having greater availability of co-located medical and mental health services (77.9% compared to 36.3% at KPNW, χ^2^ = 44.29, *p* < .001). In addition they were more likely to report that co-located medical and laboratory services facilitated prevention efforts (mean = 2.47, SD = .83 in CHCs; mean = 2.23, SD = .95 at KPNW, F(1236) = 4.03, *p* = .046).

### Characteristics that predict preventive services

To assess which variables influenced care, we first estimated variance in care-gap rates, within clinicians, separately for each sample using a fully unconditional model. There was sufficient variability within clinicians in both the KPNW sample (τ_00_ = .03, *p* < .001) and in the CHC sample (τ_00_ = .04, *p* < .001) that could be explained by patient predictors. The intraclass correlation, or proportion of variance in care gaps between clinicians, was 2.9% for KPNW and 3.8% for the CHCs. Put another way, less than 4% of the variance in care-gap rates was explained by clinician factors; the larger remaining proportion of variability was accounted for by patient-level differences.

Next we combined all of the provider-level predictors with significant bivariate relationships with care gaps into overall models for each of our populations (KPNW and CHC). In the KPNW sample, after controlling for patient-level characteristics, patients of female clinicians had fewer care gaps (γ= − .07, *p* = .011) than those of male clinicians. Additionally, the more likely a clinician was to report that s/he makes preventive care recommendations when patients are *not* experiencing acute mental health symptoms, the lower their patients’ care-gap rates (γ= − .05, *p* = .041). Additional results are reported in Table 2.

**Table 2:** Clinician-Level^a^ Coefficients for Provider Characteristics in Final Multi-Level Poisson Regression Models Predicting Care Gaps

Provider-Level Variables	KPNW		CHCs	
	Coeff.	*p*	Coeff.	*p*
Gender (Female)	−.07	*.011* ^b^	−.01	.754
Years practice	.00	.756	−.004	*.010* ^c^
HEDIS goals increase likelihood of services	.03	.231	−.06	.006
Likelihood of providing care absent symptoms	−.05	*.041* ^b^	−.05	*.033* ^b^
Case manager	−.02	.444	.06	*.028* ^b^
Care navigator	.03	.506	.05	.062

^a^Patient-level covariates controlled for in the model include age, gender, race, Hispanic ethnicity, comorbidity, [32] number of primary care visits, number of non-primary care, Medicare status and Medicaid status. For frequencies and further details on patient-level covariates, please see Yarborough et. al. [9]

^b^p < .05

^c^p < .01

Clinician gender was not a significant predictor of care gaps in the CHC sample. Consistent with the KPNW sample, however, clinicians in this sample who were more likely to report providing care to patients when they were not experiencing mental health symptoms had patients with fewer care gaps (γ= − .05, *p* = .033). Additionally, CHC clinicians who stated that performance goals increased their likelihood of providing preventive care also had patients with lower care-gap rates (γ= − .06, *p* = .006) as did clinicians who had practiced longer (γ= − .004, *p* = .010). Unexpectedly, having a case manager available in the practice was associated with having higher care-gap rates (γ=.06, *p* = .028). No other clinician or organizational characteristics were associated with care-gap rates. Additional results are reported in Table 2.

Given the relationship between patient care-gap rates and clinician-reported likelihood of making preventive care recommendations when patients were not experiencing significant mental health symptoms, we conducted post-hoc analyses to see which clinician characteristics and beliefs were associated with clinician response to this survey item. Being female (b = .242, *p* = .001), and believing that the stability or instability of patients’ mental health did not influence their own prevention-related behavior (b = .083, *p* = .019), were both positively related to reporting making recommendations to psychiatrically asymptomatic patients. By contrast, self-reported comfort interacting with patients experiencing serious mental illness symptoms (b = .000, *p* = .991), knowledge of how mental health problems and how their treatment affects physical health (b = −.057, *p* = .324), years in practice (b = .000, *p* = .988), and experience with these patients (b = −.047, *p* = .446) did not predict clinicians’ ratings of their likelihood to make recommendations to asymptomatic patients.

## Discussion

This study linked clinician and organizational predictors of preventive service delivery among individuals with mental illnesses to observed preventive service outcomes. We found that clinicians of patients with lower preventive care-gap rates reported that they were highly likely to make preventive care recommendations to patients when they were psychiatrically asymptomatic. These clinicians were also more likely to be female and to report that the stability or instability of their patients’ mental health did not affect their approach to preventive care. Put another way, these mostly female clinicians may have believed that they were equally likely to make recommendations whether their patients had active symptoms or not, and they had the patients with the lowest care gap rates. Patients of female clinicians in the KPNW sample had lower care gaps than patients of male KPNW clinicians overall. Potential practice differences between female and male physicians may have important clinical implications for patient outcomes. There is evidence that, compared to male physicians, female physicians may be more likely to systematically advise patients about prevention [29], adhere to guideline recommendations [32], and provide higher quality care [33–36].

While KPNW clinicians were more likely than CHC clinicians to report an organizational emphasis on care quality and to believe that quality goals increased likelihood of providing preventive care, the latter belief only predicted actual reduced care gaps in CHC clinics. It is possible that population characteristics moderate the effectiveness of performance goals. For example, a substantially higher proportion of CHC patients are economically disadvantaged, homeless, Medicaid beneficiaries, or under- or uninsured compared to KPNW members [9], potentially making timely preventive screening harder to achieve. Indeed, rates of guideline-concordant care tend to be lower among Medicaid beneficiaries compared to the U.S. general population [37]. Performance goals and supports to achieve them may be more powerful for clinicians working with patients in these circumstances.

The finding that access to case managers predicted higher care gaps in the CHC settings is inconsistent with past research demonstrating the positive benefits of case managers in preventive medicine settings [38]. However, as suggested above, it is possible that this finding also reflects the nature of the CHC patient population. In health care settings, case managers are often invoked to help manage complex situations that interfere with adequate medical care (e.g., homelessness). It may be that the need for access to case managers is an indicator of greater unmet needs of the CHC populations and what appears to be poor preventive care may actually be the necessary prioritization of competing demands. It is also possible that unlike the trained case managers in the study of integrated preventive care cited above [38], case managers in the CHCs had less clear roles and diffusion of responsibility resulted in care gaps.

Interestingly, few of the hypothesized clinician and organizational predictors were associated with care-gap rates. For example, clinician perception that patients with serious mental illnesses are uninterested in prevention has been documented in previous studies [15, 18, 20], however, we found no relationship between endorsing this belief and actual care gaps. Neither did we observe relationships between care-gap rates and an organizational emphasis on care quality; use of team-based care models; availability of care navigators; amount of mental health consultation; or co-location of services. This is consistent with prior work suggesting that patient characteristics are more strongly associated with screening completion than clinician or practice characteristics [20].

### Limitations and strengths

Generalizability of our findings is limited to individuals with at least minimal health services use because of our decision to require at least one health care visit for inclusion in the analyses of care gaps. We were unable to determine the representativeness of our clinician sample because we could not collect data on non-responders. However, more than half of the clinicians we contacted returned a completed survey, a rate substantially higher than reported in other studies linking clinicians to patient outcomes, enabling a much larger empaneled patient sample [39]. The KPNW and CHC settings were purposefully chosen for their differing health care delivery models, including a private health plan and publicly funded safety-net clinics, and because of the economic, geographic, and racial/ethnic diversity of each setting’s population characteristics.

## Conclusions

Clinicians who report they are likely to address preventive concerns when seriously mentally ill patients present without apparent mental health symptoms had patients with fewer care gaps. Clinician performance goals related to care quality may facilitate preventive care, especially in CHCs. Availability of case managers in CHCs was unexpectedly associated with greater preventive service gaps, warranting additional study.

## Additional file

Additional file 1: Physician questionnaire. (PDF 51 kb)

## Abbreviations

BMI: Body mass index
CHC: Community health centers
EHR: Electronic health record
KPNW: Kaiser Permanente Northwest
