# Supplementary material for: Effects of primary care clinician beliefs and perceived organizational facilitators on the delivery of preventive care to individuals with mental illnesses
Source: BMC Fam Pract. 2018 Jan 12;19:16. doi: 10.1186/s12875-017-0693-2 (PMC5767018; doi:10.1186/s12875-017-0693-2)
Supplement: Additional file 1: — Physician questionnaire. (PDF 51 kb) [file 12875_2017_693_MOESM1_ESM.pdf]

Physician Questionnaire

[The survey was formatted to be collected online using survey software]

Thank you for agreeing to tell us about your experiences providing preventive medical services to individuals with and without mental illnesses. In this survey, *serious mental illness* refers to schizophrenia, bipolar disorder, and other psychotic disorders. A few items ask you to think about those with mental illnesses who are experiencing symptoms versus those who are not. We sometimes use "other" as a shorthand way of describing your general patient panel (i.e., those who do not have serious mental illnesses).

1. How would you characterize the amount of experience you have working with patients who have serious mental illnesses?
   A great deal
   A moderate amount
   A little
   None at all
2. How would you characterize your knowledge of how serious mental health problems, and their treatment, affect physical health?
   Extremely knowledgeable
   Very knowledgeable
   Moderately knowledgeable
   Slightly knowledgeable
   Not at all knowledgeable
3. How comfortable are you interacting with patients who are experiencing serious mental health symptoms (e.g., delusions, hallucinations, symptoms of mania etc.)?

   Extremely comfortable
   Very comfortable
   Moderately comfortable
   Slightly comfortable
   Not at all comfortable

4. How concerned are you that health-related recommendations you make to patients with serious mental illnesses could potentially destabilize their mental health (e.g., suggestions to stop smoking, lose weight, etc.)?
   Extremely concerned
   Very concerned
   Moderately concerned
   Slightly concerned
   Not at all concerned
5. How much does the stability or instability of your patients' mental health affect whether or not you recommend preventive services?
   A great deal
   A moderate amount
   A little
   Not at all

6. Compared to your other patients, how much do you think your patients with serious mental illnesses care about their preventive health care?
    - They care a great deal more than my other patients
    - They care slightly more than my other patients
    - They care about the same amount as my other patients
    - They care slightly less than my other patients
    - They care a great deal less than my other patients
7. Compared to your other patients, <u>how much additional support</u> do your patients with serious mental illnesses need to be successful with preventive health care?
    - A great deal more
    - A moderate amount more
    - A little more
    - No additional help (they are the same as my other patients)
8. When your patients with serious mental illnesses are **<u>not</u>** experiencing significant mental health symptoms, how likely are you to make recommendations about the following?

	Extremely likely	Very likely	Somewhat likely	Not at all likely
Smoking				
Diet/nutrition				
Exercise				
Alcohol/drug use				
Getting a mammogram				
Getting laboratory work				
Getting a flu shot				
Completing a colon cancer screening				

9. We know that the presence of acute mental health symptoms can affect the interaction and topics covered during a visit. How much <u>less likely</u> are you to make recommendations about the following when your patients with serious mental illnesses are **<u>experiencing</u>** acute symptoms<u>?</u>

	A great deal less likely	Somewhat less likely	I would still try to talk about this
Smoking			
Diet/nutrition			
Exercise			
Alcohol/drug use			
Getting a mammogram			
Getting laboratory work			
Getting a flu shot			
Completing a colon cancer screening			

10. When making recommendations to your patients who have serious mental illnesses about behavior changes needed to improve their health, how confident are you that they will follow through?
    - Extremely confident
    - Very confident
    - Moderately confident
    - Slightly confident
    - Not at all confident
11. When making recommendations to your other patients about behavior changes needed to improve their health, how confident are you that they will follow through?
    - Extremely confident
    - Very confident
    - Moderately confident
    - Slightly confident
    - Not at all confident
12. Thinking about your patients with serious mental illnesses, to what extent do you agree with each of the following statements?

	Strongly agree	Somewhat agree	Neither agree nor disagree	Somewhat disagree	Strongly disagree
a. Smoking cessation counseling with these patients is an effective use of my time as a clinician					
b. They try to change their lifestyles if I advise them to do so					
c. Health education does little to promote these patients' adherence to a healthy lifestyle					
d. These patients use smoking to help regulate their symptoms					
e. I am less effective than professional counselors in getting these patients to quit smoking					
f. They will take my advice to lose weight					
g. They will rarely change their behavior on the basis of my advice					
h. These patients are just not ready for preventive care and therefore I don't focus on it					

13. How much do you consult with mental health providers about the physical health of your patients with serious mental illnesses (these could be both clinicians who prescribe medications and those who do not)?
    - A great deal
    - A moderate amount
    - A little
    - Not at all

14. How satisfied are you with the level of coordination you have with mental health providers regarding your patients with serious mental illnesses (these could be both providers who prescribe medications and those who do not)?
    - Extremely satisfied
    - Very satisfied
    - Moderately satisfied
    - Slightly satisfied
    - Not at all satisfied
15. How helpful do you think it would be for mental health providers to talk about the importance of preventive health care with their patients who have serious mental illnesses?
    - Extremely helpful
    - Very helpful
    - Moderately helpful
    - Slightly helpful
    - Not at all helpful
16. How helpful do you think it would be for mental health <u>medication prescribers</u> to order or provide basic preventive care services (e.g. blood pressure, weight, lab work) as part of mental health visits?
    - Extremely helpful
    - Very helpful
    - Moderately helpful
    - Slightly helpful
    - Not at all helpful
17. Has your clinic adopted any of the following approaches to care delivery? (check all that apply)

    a) Medical or health home
    b) Co-location of medical and mental health services
    c) A team-based care model
    d) Behavioral health specialists
    e) Case management
    f) Care navigators (people who go into the community to help patients access and use health services and the health care system)
    g) Assertive Community Treatment (ACT)
    h) Health coaches
    i) Peer-provided services
    j) Community outreach services
    k) Other (please specify):____________________________

18. How much does each of the following *facilitate* the delivery of health promotion and disease prevention services to patients with serious mental illnesses in your practice?

	A great deal	A moderate amount	A little	Not at all	Not applicable or not available
a. The use of mobile prevention services (e.g. mobile mammography van)					
b. Co-location of laboratory services					
c. Co-location of mental health services					
d. Co-location of more complicated preventive services (e.g. on-site mammogram or colonoscopy services)					
e. A medical record system that helps you/your staff remind and prompt patients about preventive services					
f. Team-based care					
g. Having someone "scrub" records to identify patients who need preventive services, either for outreach or prior to visits					

19. How much does each of the following potential *barriers* affect the delivery of health promotion and disease prevention to the patients with serious mental illnesses in your practice?

	A great deal	A moderate amount	A little	Not at all
a. Lack of time				
b. Insufficient reimbursement for preventive services				
c. Lack of systems for prompting and tracking preventive services				
d. Lack of patient interest in prevention				
e. Communication difficulties with patients				
f. Cultural differences between you and the patient				
g. Patients come in with acute problems				

20. To what degree does your group practice emphasize the following?

	Not at all	A little	A moderate amount	A great deal
a. A focus on cost-effectiveness				
b. Patient satisfaction				
c. A patient-centered approach to care				
d. The cost-effectiveness of treatment protocols				
e. Quality of care				
f. Staying on time with appointments				

21. To what extent do preventive screening goals and measures (e.g., HEDIS, Uniform Data System, or other quality measurement, state or federal reporting requirements) affect the likelihood that you will deliver preventive care for individuals with serious mental illnesses in your practice?

Greatly increases the likelihood
Somewhat increases the likelihood
Has no effect on the likelihood
Somewhat decreases the likelihood
Greatly decreases the likelihood

22. Please describe any unintended negative consequences of collecting HEDIS or other performance goals and measures that affect the way services are delivered (e.g., less likely to record problems if it means the HEDIS measure requires follow-up, etc.) [open-ended]

23. Please describe how the new CMS reporting system (Physician Quality Reporting System) affects your plan to deliver preventive services to patients with serious mental illnesses, if at all. [open-ended]

24. Please describe any advice you have for other clinicians about how to approach providing preventive services to people who have serious mental illnesses? [open ended]

25. We know that clinicians are under a lot of pressure. Sometimes this and other difficulties can lead to burnout. Using your own definition of burnout, please tell us how you feel about your work generally:

   a. I enjoy my work. I have no symptoms of burnout.
   b. Occasionally I am under stress, and I don’t always have as much energy as I once did, but I don’t feel burned out.
   c. I am definitely burning out and have one or more symptoms of burnout, such as physical and emotional exhaustion.
   d. The symptoms of burnout that I’m experiencing won’t go away. I think about frustration at work a lot.
   e. I feel completely burned out and often wonder if I can go on. I am at the point where I may need some changes

26. How many years have you been practicing medicine (after finishing clinical training?)

27. What is your gender?

28. What is your age?

29. Are you Latino or Hispanic? Yes/no

30. How would you describe your race or ethnicity? (check all that apply)

White or Caucasian
African American or Black
Asian or Pacific Islander or Native Hawaiian
American Indian, Native American or Alaska Native
Other, please specify:________________________________
